# Applying the extended attitude formation theory to central bank digital currencies

**DOI:** 10.1007/s12525-023-00638-3

**Published:** 2023-04-27

**Authors:** Frédéric Tronnier, David Harborth, Patrick Biker

**Affiliations:** 1 Goethe-University Frankfurt, Theodor-W.-Adorno-Platz 4, 60323 Frankfurt am Main, Germany; 2 Capgemini Invent, Mainzer Landstraße 178 – 190, 60327 Frankfurt Am Main, Germany; 3 Ebner Stolz, Holzmarkt 1, 50676 Cologne, Germany

**Keywords:** Central bank digital currency, Attitudes, CBDC, Digital euro, Attitude formation theory, Technology adoption, O33, G41

## Abstract

This work analyzes the attitude formation process of individuals for central bank digital currencies (CBDCs), specifically for the digital euro. CBDCs are heavily researched, with pilot projects being conducted worldwide. Following the emergence of cryptocurrencies and a decrease in cash usage for retail transactions, CBDCs are viewed as the possible payment solution of the future. Using a qualitative approach, we conduct expert and non-expert interviews to apply and extend existing research on attitude formation to study how individuals form attitudes towards a CBDC in Germany. We find that individuals form their attitudes towards a digital euro through the perceived benefits, limitations, and concerns regarding related payment solutions, moderated through the perceived equivalence of these related technologies and the CBDC. The results contribute to the literature on CBDC and can be used by practitioners to develop a digital euro that offers a competitive advantage for retail transactions over established payment solutions.

## Introduction

Throughout history, money has been an essential factor in the lives of individuals while undergoing constant change (Rothbard, 1993). Money evolved from physical coins and paper-based banknotes to credit cards and digital means of payment. Right now, the financial and monetary system is facing the next, possibly revolutionary, change in money with the introduction of digital currencies (European Central Bank, 2020) and, at the same time, a decrease in physical cash payments, especially in developed countries, such as Sweden (Sveriges Riksbank, 2020).

Central banks reacted to these challenges by discussing the implementation of central bank digital currencies (CBDC). Various central banks are researching and piloting CBDC solutions for both interbank payments and settlements as well as general purpose solutions, intended to be used as digital cash equivalents for end users (Tronnier et al., 2020) with “central banks collectively representing a fifth of the world’s population […] likely to issue a general purpose CBDC in the next three years” (Boar et al., 2020).

The European Central Bank (ECB) is presently investigating the introduction of a digital euro and has conducted a survey among individuals, experts, and organizations on the perception of stakeholders of such a digital euro. Through the ECB’s consultation among 8221 respondents, EU citizens have already clearly articulated their preference for a privacy-sensitive and secure solution that offers usability across the whole euro area (European Central Bank, 2020). The objective of the ECB regarding the digital euro is clearly stated by ECB president Christine Lagarde: “Our role is to secure trust in money. This means making sure the euro is fit for the digital age” (European Central Bank, 2020). Presently, little is known about the digital euro’s final properties yet, except basic requirements initially laid out by the ECB: the digital euro is a digitalized representation of the existing euro that will be introduced alongside cash. It shall be usable by every citizen and might exhibit cash-like features (European Central Bank, 2020).

Current research on CBDC focuses on the implementation of technical features (Gross et al., 2021) or discusses potential advantages and disadvantages of CBDC for the economy or central banks (Sidorenko & Lykov, 2019). Research that focuses on the future user of a CBDC is scarce, which is surprising as the success or failure of the implementation of a CBDC will ultimately depend on users’ attitudes towards a CBDC and their usage of it. Although first research on CBDC adoption already exist (Solberg Söilen & Benhayoun, 2022), it is crucial to first focus on the process through which individuals form attitudes towards CBDC. Attitudes may be defined as the feelings of an individual towards a particular behavior or technology (Ajzen & Fishbein, 1980). Existing models that study technology acceptance such as the unified theory of acceptance and use of technology model (UTAUT) are only usable if there is at least a mature imagination of users about how the technology in question works. We argue that for CBDC, users are currently unable to evaluate factors such as effort expectancy or ease of use as CBDCs have largely not been implemented yet. Technical features and design considerations are not determined yet and differ significantly between published reports of differing central banks. Moreover, previous research has shown that known theories related to the adoption of technological innovations, e.g., utility theory, cannot always be adapted towards cryptocurrencies and blockchain technology, a digital payment technology related to CBDC, in a one-to-one manner (Esmaeilzadeh et al., 2019). In this work, we therefore specifically focus on attitudes towards a digital euro, as they are a predecessor of behavioral intentions and ultimately actual use (Ajzen & Fishbein, 1977; Fishbein & Ajzen, 1977). As a theoretical framework, we adapt the model of the extended attitude formation theory (EAFT) (Harborth & Kreuz, 2020), which postulates that attitudes towards new technologies are influenced by existing beliefs about related technologies and the perceived equivalence between those technologies.

Thus, this work aims to close the identified research gap by answering the following research question:

Which factors influence end users’ attitudes towards central bank digital currency (CBDC)?

Since the digital euro is a new financial technology with the previously described lack of concrete features, we need to address our research question using an approach that considers two points. First, we argue that the research is exploratory by nature, thus requiring a qualitative research method. There is, to the best of our knowledge, no prior research on individuals’ attitudes towards CBDCs, which constitutes a first step towards the study of actual CBDC adoption and usage. Second, we argue that it is not beneficial to solely rely on data from layman users for addressing our research question as experts on the topic of digital currencies and CBDCs might provide additional insights otherwise left uncovered. Therefore, we conduct semi-structured interviews to obtain data from laymen users as well as experts on the topics of CBDCs, the digital euro, and related established payment methods and technologies. The data is evaluated using qualitative content analysis (Mayring, 2015) to create an adapted EAFT model (Harborth & Kreuz, 2020) that demonstrates that existing attitudes towards other payment methods influence beliefs about CBDC. We decided to use the EAFT and the attitude formation process as this enabled us an exploratory derivation of CBDC-specific factors without being tied to existing concepts from technology adoption literature as one of our main goals is to find currently unknown factors which are relevant for individuals when forming their attitudes towards CBDC.

The resulting model and analyzed findings demonstrate that attitudes towards a digital euro are indeed formed and influenced by respondents’ perceptions about related payment methods and technologies, as well as through payment context- and user-specific factors.

Thus, we contribute to the literature on financial technology by applying the extended attitude formation theory (EAFT) to the case of the digital euro with the qualitative data generated through our interviews. The findings demonstrate the close linkage between attitudes towards existing payment solutions and the digital euro from an individuals’ perspective. Attitudes towards a digital euro are formed through the perceived equivalence of established solutions with the digital euro, as well as through payment context- and user-specific factors.

## Central bank digital currency and the digital euro

The digital euro is intended to act as a currency based on the three main economic functions of a currency (Yermack, 2015). First, as a medium of exchange, it enables trade between two parties by allowing the exchange of goods and services for the currency. Second, as a unit of account, it enables price setting and facilitates comparability of costs for products and services. Third, as a store of value, it preserves its value over time.

In order to define CBDCs, it is necessary to differentiate between different types of money based on their main properties: issuer, form, accessibility, and technology (Bech & Garratt, 2017). The issuer may be a central bank or other private banks, and money can be digital or physical and may be accessible to the general public or limited to interbank transactions. Lastly, money can be account-based or, in the case of distributed ledgers or blockchains, token/value-based. Presently, only banknotes and coins are issued by central banks as legal tender for the general public, which represents a liability to the central bank (Kiff et al., 2020). Bank deposits on the other hand are issued by private commercial banks and thus do not constitute a legal tender. The digital euro can therefore be defined as a digital form of central bank-issued money for the general public, with no decision made yet on its underlying technology.

In accordance with the information provided by the ECB on the digital euro (European Central Bank, 2020), this work focuses on the digital euro as a retail CBDC that is to be introduced as an accessible form of digital money for the general population in the euro area. The proclaimed objective is to facilitate online retail payments, with the digital euro not replacing cash, although an offline functionality for contactless payments is envisioned as a desired feature (European Central Bank, 2020). As most CBDCs have not been available for the general public yet, concrete technological features differ strongly between the reported approaches of various central banks. For the digital euro in Europe, a plethora of central bank research investigates different design choices. For instance, the transfer of digital euros could be conducted using software and smartphone applications or through the use of a hardware bearer instrument (HBI). Such a HBI, a hardware device, is investigated by the German central bank (Deutsche Bundesbank, 2021) as part of multiple workstreams on a digital euro by the Eurosystem, the organization that incorporates all national EU central banks. The work concludes that a HBI would be technically feasible and could allow for transactions between two parties, without the need for an intermediary, offering a close resemblance to cash. However, a HBI solution offers security concerns and is currently unable to prevent that digital euro could be brought and spent in countries and regions that the Eurosystem does not intend to offer digital euro to. Apart from the question whether to use a hardware- and/or software-based solution for digital euro transactions, research is discussing the use of distributed ledger, or blockchain, technology on the one hand and the use of an account-based model on the other hand. Based on the public consultation on the digital euro (European Central Bank, 2020, 2021), the central bank of Italy investigates this in more detail and advocates for an integrated model that combines the advantages of both account- and token-based models (Urbinati et al., 2021). The authors argue that some of the desired features, in particular cash like privacy, can only be achieved using a token-based approach, while many others, including compliance with AML/CFT requirements, can only be achieved using an account-based approach. While these technical design choices are not made yet, the core principles for the introduction of a CBDC in Europe are clearly communicated: the aim to not issue a parallel currency to physical cash but to issue digital, risk-free central bank money through the Eurosystem that is widely accessible in all euro countries. It is to be highly trusted by end users and should not crowd out private solutions for digital retail payments (European Central Bank, 2020). Under consideration of these principles and the current technological designs, it is likely that the actual process of conducting CBDC transactions might not change significantly for retail end users. As central banks do not want to take away retail banks’ core responsibilities, it is possible that the overall architecture for retail CBDC would follow the hybrid solution by Auer and Böhme (2020) in which CBDC would represent a claim on the central bank but central banks would not directly interact with end users. Instead, retail banks would remain acting as intermediaries by handling user onboarding, ensuring know-your-customer (KYC) requirements and conduct CBDC transactions. Central banks would remain overseeing transactions and issuing CBDC.

In recent years, academic and central bank interest in CBDCs has soared to new heights. Prior work finds that both central bank and academic research on the topic rarely consider societal aspects and implications of CBDCs (Tronnier et al., 2020). Instead, most research focuses on monetary policy and economic aspects. Additionally, design properties are frequently discussed to study the implication of certain features on the banking system. For instance, Yanagawa and Yamaoka (2019) of the Bank of Japan study the effect of an interest bearing CBDC, as well as the installation of a holding limit in CBDC. The majority of work, however, focuses on providing a comprehensive introduction to the topic and discusses the different rationales, benefits, and limitations of the introduction of CBDC in different countries (Sidorenko & Lykov, 2019; Yao, 2018). The possible adoption of CBDCs has been studied to a limited degree. From a central bank perspective, various central banks discussed possible benefits a CBDC might provide them in fulfilling their mandates. Such benefits include an increase in payment efficiency and financial stability as well as the fostering of financial inclusion and the prevention of money laundering (Barontini & Holden, 2019; Bech & Garratt, 2017; Mancini-Griffoli et al., 2018).

Other work studies CBDC from a technical perspective, for instance, by aiming to develop privacy-friendly CBDC solutions (Gross et al., 2021). Privacy and trust are factors often researched in the context of CBDCs, e.g., Atako (2020) advocates for a privacy framework for retail CBDCs by reviewing the US Privacy Act of 1974 and proposes refining it to maintain a balance between privacy and transparency in digital payments. Trust is seen as a requirement for the acceptance of money which needs to be extended towards CBDCs (European Central Bank, 2020; Lee et al., 2021; Patel & Ortlieb, 2020). The Official Monetary and Financial Institutions Forum (OMFIF) published a survey on trust in digital currencies in 2020, showing that central banks are the entity most trusted in issuing a digital currency (Patel & Ortlieb, 2020). Using only a very limited number of questions, the study finds that attitudes towards payment options depend on the demographic characteristics of respondents, with respondents from emerging markets being more likely to embrace digital currencies compared to respondents in developed countries.

Another work by the Dutch central bank studies the possible adoption of CBDCs using a Dutch consumer panel and finds that price incentives, trust in banks and central banks, privacy protection, and knowledge about CBDCs are important drivers for CBDC adoption (Bijlsma et al., 2021). This work differs from ours as it does not follow an established scientific procedure but surveys individuals’ preferred features for a CBDC.

## Related work and theoretical foundation

In the following chapters, related work on adoption and attitudes are discussed upon which the theoretical model of this work is build.

### Related work on adoption and attitudes

The information systems domain has focused strongly on researching the adoption of new technology in the past. Digital payment methods, such CBDC, are regarded as a new technology that is to be used by end users in the future. Given to close link between adoption and attitudes (Ajzen & Fishbein, 1980), we firstly discuss related research on technology and digital payment adoption before reviewing related work on attitudes towards technology.

Many known models from information systems literature, such as the technology acceptance model (TAM) (Davis, 1989) or the unified theory of acceptance and use of technology, UTAUT and UTAUT2 model (Casquejo et al., 2020; Venkatesh et al., 2003, 2012), have been applied to study payment systems and cryptocurrency adoption. Most models have been adjusted by adding external factors such as trust (Lu et al., 2011), perceived security (Ramos-de-Luna et al., 2016), or privacy concerns (Mutahar et al., 2018; Su et al., 2018). Ramos-de-Luna et al. (2016) adapted a model based on TAM to study attitudes towards NFC payments, identifying the factors perceived usefulness, compatibility, security, and subjective norms to significantly influence attitudes. The UTAUT2 model was used by Alalwan et al. (2017) and Kim and Bae (2020) to study mobile banking adoption. Technology adoption has also been studied for past payment-related innovations, such as ATM adoption by banking firms (Hannan & McDowell, 1984; Sharma, 1991). With regard to attitudes, Dang et al. (2022) study antecedents and outcomes of attitudes towards facial recognition payment solutions. Using a Chinese sample of individuals that have already used such technology, the authors find that factors such as perceived ease of use significantly influence attitudes that in turn influence store satisfaction. Other research that studies attitudes is using the aforementioned TAM model to, in fact, study technology adoption for mobile payment services in a Swedish sample (Arvidsson, 2014). Again, factors such as ease of use are found to be significant antecedents of intention to use. However, the author notes that “studies of innovation in the payment industry cannot rely on TAM and innovation diffusion theory alone” (Arvidsson, 2014, p.1).

The central bank of the Netherlands conducted a survey with 2522 participants on adoption intentions of a CBDC and finds that trust in banks and central banks, as well as knowledge on CBDC, increases adoption intention (Bijlsma et al., 2021). However, the work does not utilize a specific model but instead relies on regressions to determine the effect of factors on adoption intention. Bai (2020) combines the UTAUT2 and the Theory of Planned Behavior (TPB) (Ajzen, 1991) to study the adoption of digital currencies, whereby digital currencies include both cryptocurrencies and an undefined CBDC. The author finds the construct price value and perceived behavior control to be insignificant. Solberg Söilen and Benhayoun (2022) adapt the UTAUT model to a CBDC and analyze it through a quantitative survey. The authors integrate trust in the currency system into their model and aim to assess actual use behavior through their questionnaire. We argue that it is not possible to study actual use behavior through a survey, especially given the fact that CBDCs have not been available for respondents yet. Moreover, we argue that it is essential to focus on a specific kind of CBDC as central banks worldwide differ greatly in their approaches for CBDCs. This is likely to result in many different types of CBDCs based on the chosen conceptual and technical design and the desired features a CBDC might possess. In the work of Tronnier et al. (2022), the authors study privacy concerns in CBDC by adapting the antecedents, privacy concerns, and outcomes (APCO) model by Smith et al. (2011). Using a survey, the authors find that several antecedents such as self-efficacy and perceived vulnerability as well as soft trust factors influence privacy concerns. Privacy in CBDC has also been studied using a qualitative approach, identifying several antecedents and factors that contribute to privacy concerns (Tronnier & Biker, 2022).

The work conceptually closest to ours is a qualitative analysis of adoption factors for stablecoins (Kimmerl, 2020). The author conducts 32 interviews to study adoption factors of stablecoins, such as the now stopped Diem project initially proposed by a payment consortia led by Meta. Using grounded theory, seven primary themes and 43 sub-themes are described which influence adoption intentions for stablecoins.

In IS, technology adoption and usage behavior are closely linked to attitudes towards technology. In the theory of reasoned action (TRA), attitudes relate to the feelings of individuals towards a particular behavior (Ajzen & Fishbein, 1980). A favorable outcome of a behavior will hereby lead to positive attitudes towards said behavior, while unfavorable outcomes relate to negative attitudes. Later, through the introduction of the UTAUT, more recent research did not include attitudes in their models. For the UTAUT, Venkatesh et al. (2003) stated that attitudes are only found to be significant when newer “constructs related to performance and effort expectancies – are not included in the model” (Venkatesh et al., 2003). Kai-ming and Enderwick (2000) studied individuals’ attitudes in the manufacturing context and define six beliefs which contribute to technology adoption: compatibility, enhanced value, perceived benefits, adaptive experiences, perceived difficulty, and suppliers’ commitment. The authors define attitudes as “the cognitive process which depicts the prospective adopter’s positive or negative affection about adopting a foreign technology” (Kai-ming & Enderwick, 2000, p.267).

Regan and Fazio (1977) demonstrated that the consistency between attitudes and behavior are influenced by the attitude formation process. That is, individuals who could form their attitude towards an object based through direct interaction with the object demonstrate greater consistency between attitudes and behavior, as opposed to individuals who formed their attitudes through other means. To the best of our knowledge, no research studying attitudes towards CBDCs specifically has been conducted. However, past research on former new payment methods, such as (credit) cards and payment-related services such as ATMs, exists. For instance, early adopters of credit cards and ATMs were found to be on average younger (Bank Marketing Association, 1977 as cited by Swinyard and Ghee (1987)), living in higher populated cities (Hirschman and Julander, 1979) and had more positive attitudes towards these new payment technologies in general (Awh & Waters, 1974). For ATMs specifically, Rugimbana (1995) showed that individuals’ perceptions towards the technology, such as expected convenience, are a much stronger predictor of usage than demographic variables. Murdock and Franz (1983) find that habits and perceived risks significantly correlate with resistance to use ATMs.

In summary, our study of related work demonstrates that it is necessary to investigate user attitudes with regard to the digital euro in greater detail. Prior work focuses on technology adoption using existing models that are adapted by adding new factors, even as CBDCs cannot yet be truly experienced by individuals.

Thus, we argue that the analysis of attitudes constitute an important first step towards understanding the adoption of CBDCs. In addition, prior work indicates that it is often not possible to assume that existing models can be applied on new technologies as individuals are not able to assess critical factors such as usability or performance without any actual experiences (Harborth & Kreuz, 2020). The authors also argue for the inclusion of attitudes towards known technologies that are comparable by individuals to the one in focus. Harborth and Kreuz (2020) investigate user attitudes towards augmented reality (AR) using grounded theory and identify the relationships of perceived attitudes towards related technologies, that respondents already have experienced, as important factors in the attitude formation process. As a digital euro has not been implemented yet, it is not trivial for individuals to assess factors present in existing models such as TAM or UTAUT, e.g., the perceived ease of use and performance expectancy of a digital euro. This holds true especially as the actual performance and ease of use are likely to depend on the implementation and technical features of a digital euro which have neither been designed nor decided upon yet.

Nonetheless, we argue that individuals are already able to form attitudes on a digital euro since they were, for instance, able to answer the public consultation of a digital euro by the ECB and other central banks. Following prior, these attitudes are likely to influence an individuals’ intention to use a digital euro (Venkatesh et al., 2003).

### Theoretical model

Information systems (IS) research has extensively studied technology acceptance and attitude formation in the past. However, we argue that models such as the TAM or UTAUT are not directly applicable to the digital euro since it cannot solely be regarded as a technology but must also be regarded as a currency. There might exist additional factors which are not included in the original models. We seek out to explore all possible factors with our qualitative research approach. More importantly, since the digital euro is to be regarded as a currency, there already are other solutions—cash, credit cards, and digital as well as mobile payment solutions—against which the digital euro will need to compete. Individuals will already have formed attitudes towards these payment solutions and currencies and decided for or against their usage. Thus, we argue that we need to consider two points. First, it is necessary to investigate citizens’ attitudes towards CBDCs as a first step before assessing adoption intentions. Second, we hypothesize that attitudes towards CBDCs are largely influenced by individuals’ perceptions regarding known alternatives (Harborth & Kreuz, 2020), against which CBDC competes as the payment solution of choice.

However, the problem is that technology acceptance models have in common that attitudes and beliefs are only considered for the technology in focus of the particular research. Thus, attitudes towards other or related technologies are not considered in these models. The only paper—to the best of our knowledge—which considers the effects of known and related technologies on the attitude formation process of innovations is the article by Harborth and Kreuz (2020). The authors study attitudes towards augmented reality (AR) technologies using grounded theory through 12 interviews and find that attitudes towards AR are formed based on the perceived benefits, limitations, and concerns of AR technologies. These categories, in turn, are influenced through perceived benefits, limitations, and concerns of technologies that individuals compare AR to, namely smartphones, computers, and 2D screens. These technologies are already known to individuals, and attitudes towards them are found to influence attitudes towards AR. The perceived equivalence of these technologies to AR serves as a moderator on the relationships. The authors generalize their model through the combination of their empirical results with existing literature on attitudes into the extended attitude formation theory (EAFT). This theory states that attitudes towards new technologies are influenced by existing beliefs about related technologies and their perceived equivalence with the innovation in focus. The theory is conceptually linked to the trust-transfer model of Stewart (2003) that finds that trust on the internet is transferred between websites through hyperlinks. However, the EAFT does not focus on the factor of trust alone but considers attitudes towards a technology in general. We therefore apply EAFT and adapt it towards the context of CBDC to form our preliminary research model that is then evaluated and refined through the analysis of the interviews. We argue that this gives us the opportunity to overcome the limitations of other models, such as the TAM, as its factors perceived ease of use or performance expectancy cannot be adequately assessed by individuals, given that they were not able to actually interact with and use CBDCs yet.

**Fig. 1:**
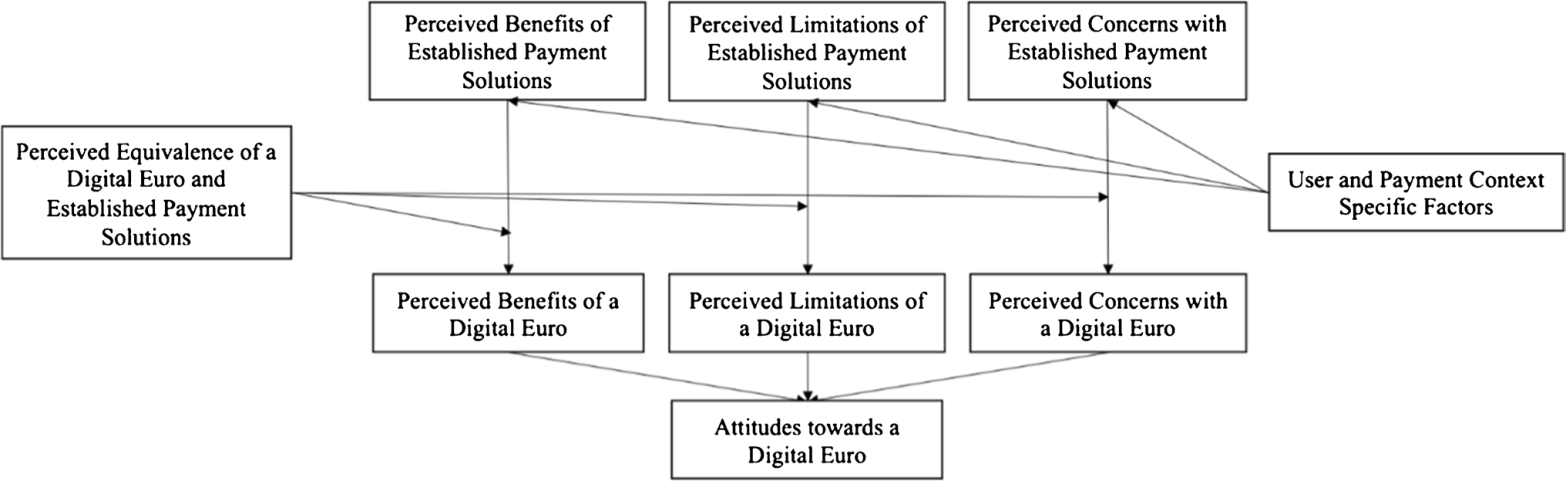
Research model on attitudes towards a digital euro, adapted from Harborth and Kreuz (2020)

The resulting model is depicted in Fig. 1 and its factors explained in the following chapters.

## Methodology

In their work on grounded theory, Glaser and Strauss (1967) state that “generating a theory from data means that most hypotheses and concepts not only come from the data, but are systematically worked out in relation to the data during the course of the research. […] By contrast, the source of certain ideas, or even ‘models,’ can come from sources other than the data” (Glaser & Strauss, 1967, p.6). We follow this approach by conducting qualitative research to study end user attitudes towards a digital euro. In contrast to quantitative research, the purpose of our qualitative research approach is not to discover statistically significant correlations of variables, but to investigate a research object in an exploratory manner. The “model” (Glaser & Strauss, 1967) upon which we build our research is the attitude formation theory (EAFT) by Harborth and Kreuz (2020).

Lee and Baskerville (2003) and Yin (2003) argue that there exist different forms of generalizability, apart from the statistical generalization. With this work, we do not aim to validate our model but to generalize from descriptions, that are the statements made by respondents, to form a theoretical model. The result is not expected to be a proven statement but a “well-formed hypotheses” (Lee & Baskerville, 2003, p. 224).

As research on factors that might influence user attitudes towards CBDC is scarce, we conduct a qualitative content analysis using semi-structured interviews with experts and non-experts (Mayring, 2015). This approach offers the opportunity to validate existing dimensions, identified from prior literature, and to introduce new dimensions to the model which emerge during the qualitative analysis process. Semi-structured interviews were used as they allow more room for interaction between interviewer and interviewee which ensures that the whole picture is captured (e.g., by asking follow-up questions).

Interviews with both experts and non-experts were deemed necessary because the digital euro will be an entirely new form of money and technology, designed and developed by experts to be used by ordinary citizens. Given a possible information asymmetry between these two groups, it is plausible to assume that attitudes between the groups might differ. In this work, an expert is defined as a person in academia, a private corporation, or public institution with a professional background either directly related to the digital euro or associated fields and topics of interest for this work, such as blockchain technology, cryptocurrencies, payment services, or cybersecurity and data protection.

The qualitative content analysis is implemented as structured content analysis. This form of content analysis obtains its coding categories by a deductive derivation of existing theories (Mayring & Fenzl, 2014).

### Interview guideline

The semi-structured interviews were conducted using an interview guideline. This guideline was designed to ensure a maximum of openness so that an interviewee can freely express their opinion (Helfferich, 2014). Simultaneously, it imposed the necessary structure to direct the interview towards the content required for answering the research question. Prior to the interview, interviewees were informed that they were participating in research about the digital euro but were not informed on specific questions, e.g., regarding potential concerns with existing payment solutions and a digital euro as to avoid socially desirable responding (Wetzel et al., 2016).

The guideline was designed to utilize open-ended questions about attitudes towards a digital euro and an interviewee’s expectations regarding its features. Respondents were first asked to elaborate on their actual payment behavior to encourage stating their behavior and attitudes rather than covering what the interviewee beliefs to be the “correct” answer for the interviewer or under consideration of societal norms.

The interview guideline is structured as follows: After a brief introduction, the interviewee’s current use of digital payment methods and their opinions about them is surveyed. As a digital euro is not yet available, the current usage of digital payment methods is considered to be the closest proxy to capture actual usage behavior. Next, the digital euro is introduced by a written description derived from ECB’s own definition (European Central Bank, 2020). The description of a digital euro used in this work deviates from the ECB’s definition only in that it chooses one implementation option: it is implemented as a hybrid CBDC, where the digital euro is issued by the central bank but distributed to the retail users by traditional banks rather than by the ECB directly. This specification was added to the ECB’s definition to make it easier for non-experts to imagine how a digital euro could look like. For expert interviews, this specification is intended to ensure that the interviewer and the interviewee apply the same definition of a digital euro. Next, questions about the possible use of a digital euro follow. The interview concludes with a question directly asking about privacy concerns in relation to the digital euro to investigate the importance of this factor in more depth. Follow-up questions were asked on all topics to give interviewees the opportunity to clarify statements or to explore topics, such as privacy concerns, which were identified in the related literature to be of potential importance with regard to CBDC, in more detail.

A test run of the interview guideline with two non-expert subjects yielded satisfactory results of the interview guideline, resulting in only minor changes to the questions on prior experience with payment methods and their underlying technological solutions.

### Data gathering

The decision for a qualitative approach was made as we aim to extend existing theory (EAFT), which relies on theoretical sampling rather than statistical one (Eisenhardt, 1989). With this aim in mind, the theoretical sample does not need to be random, but it is instead preferable to choose contrasting cases (Pettigrew, 1990). We therefore choose experts and non-experts on the topic as interview participants to obtain a wide range of differing views on the topic. This is in line with the notion of theoretical relevance by Glaser and Strauss (1967) who argue that researchers should choose any groups, in our case respondents, that can help to generate as many properties and categories as possible. Respondents should have enough in common that they are comparable and can be excluded if they demonstrate fundamental differences. Thus, the research question of this work was further specified to only investigate attitudes of German citizens, so that the exclusion criteria guarantee that respondents are experienced with the German payment market and its services. This restriction however controls for the influence of different nationalities, a factor which was found in prior research to significantly influence antecedents of attitudes and adoption intentions (Smith et al., 2011). The impact of nationality and associated culture is a factor determined by the society one is born in and is not usually a function of individual choices. The ECB consultation demonstrated that perceptions differed among European countries (European Central Bank, 2020). Accordingly, the interviews were conducted among individuals with a German citizenship or German-speaking individuals in Germany who have lived there a significant amount of time.

Experts were recruited and contacted either directly on professional social networks such as LinkedIn, through public posts in topic-related online forums, or through other means of contact. The CAC21A Crypto Assets Conference 2021 in Frankfurt, Germany, and the Digital Euro Association (DEA) were used to contact experts on the topics of cryptocurrencies, payment solutions, and the digital euro in particular. We chose the sample size based on the concept of theoretical saturation (Charmaz, 2014). This concept is the most commonly used in qualitative research and declares that the data gathering process can be terminated once no, or only very few, new insights are generated from the interviews. We used a purposive instead of a snowballing approach (Robinson, 2014) as we wanted to avoid the possibility that respondents refer us to new respondents with equal perceptions and answers on the topic. Instead, we opted to obtain expert respondents that differed from each other, i.e., researchers and industry professionals with a technical or economic background, in order to obtain as many different insights and factors as possible.

Non-experts were recruited through public posts drawing attention to this research and by spreading the word about this work on social networks. In addition, randomly selected individuals were contacted via social media and asked directly to participate to obtain insights from individuals that were not aware of the topic and likely would not have participated if not contacted directly. Non-experts therefore include pilots, actors, teachers, and multiple students that had no prior knowledge on the topic. Interviewees were not compensated for their participation in this research to exclude participants that might only be interested in the monetary benefit of participating in this study.

### Execution of the interviews

In total, 23 interviews were conducted from July until September 2021. Two interviews were counted as test interviews to test and refine the interview guide. Given the COVID-19 pandemic, interviews were conducted via individual video conferencing sessions. It is known from research on privacy in work environments that people can become anxious when they have no control over the choice of technology they use (Teebken & Hess, 2021). To ensure that the interviewees felt comfortable in the interview setting, they were able to choose the video conference solution to be used for the interview. Prior to every interview, the interviewees were informed that all their statements would be processed only under a pseudonym and personal identifiable information such as (company) names would be anonymized. We obtained consent to use the interviews for scientific purposes. From the 23 interviews conducted, only 21 were included in the final data sample. One interview was lost entirely due to a technical malfunction with Skype’s internal recording function. For expert interview no. 6, 7 min of the 28-min-long interview record were lost. It was decided to include that interview in the data sample as more than two-thirds of the interview recording were available and contained valuable information with regard to the research questions. Two other interviews with non-experts in the age group of 60 or older, however, were excluded from the data sample as both interviewees were unable to express any opinion about a digital euro and denied to answer any question related on its future use.

## Results

This section provides an overview of our sample and the categories and their relations within our research model.

### Descriptives

The final data sample has a nearly equal number of expert and non-expert interviews with ten female and eleven male participants. As the two interviews with people above 60 years were not included in the final sample, interviewees were distributed into age brackets between 20 and 59. More than 90% of the interviewees obtained some form of higher education and hold a university degree. Among the subgroup of experts, there are five interviewees with a doctoral degree in computer science or economics.

At the beginning of the interview, each participant was asked to describe whether they have any prior knowledge about the digital euro itself and two other topics initially considered to be closely related, namely cryptocurrencies and blockchain technology. Several experts did lack prior knowledge in these domains but have professional experience in the domains of finance, data privacy, law, or other fields closely linked to CBDC and the factors discussed in this work. Table 1 depicts the demographics of interviewees.

**Table 1:** Demographics

	Age				Gender		Prior knowledge											
							Blockchain tech				Cryptocurrency				Digital euro			
	20–29	30–39	40–49	50–59	Male	Female	No	Passive	Active	Advanced	No	Passive	Active	Advanced	No	Passive	Active	Advanced
NE	7	3	0	0	6	4	4	5	0	1	3	7	0	0	5	5	0	0
EX	3	4	2	2	5	6	1	3	2	5	2	1	3	5	6	1	0	4
#	10	7	1	2	10	10	5	7	2	6	4	8	3	5	10	6	0	4

Legend: *NE* non-experts, *EX* experts

Experts generally hold an advanced degree in at least one of the fields of economics, finance, business administration, law, or computer science. We define passive knowledge as knowledge acquired by experts through reading or hearing about a specific topic. Active experts are not only aware of the topic and possess some knowledge on it but are able to explain the topic to other individuals, i.e., for cryptocurrencies, active knowledge requires to have already bought or traded them in the past. Advanced knowledge depicts profound knowledge on the topic, with experts actively working in this precise field. Here, experts have, for instance, already published academic papers on the topic or developed products. There are experts with little prior knowledge on the topics depicted but with advanced knowledge on data protection and IT security that were also deemed highly relevant.

### Coding and category development

We used MAXQDA2020 for the coding, memo writing, and analytical process. We follow the structure of the deductive category assignment in a structured content analysis (Mayring & Fenzl, 2014). The procedural model is as follows: In a first step, the research question and theoretical background are defined. Our research question is to investigate individuals’ attitude formation in a digital euro. To this end, we assess related work on attitudes and adoption intention in digital payment systems and investigate suitable models that can be adapted towards the research question. In the second step, main categories and subcategories are derived from theory. Our main categories are perceived benefits, limitations, and concerns of comparable technologies or currencies, such as digital payment methods, card, and cash payments. The perceived equivalence of these technologies and currencies moderates the perceived benefits, limitations, and concerns towards a digital euro. In the third step, we develop a table that defines the main categories (Table 2).

**Table 2:** Definitions of developed categories of attitudes on a digital euro

Category	Definition
Perceived benefits of known payment solutions	Perceived positive properties of established payment solutions such as credit/debit cards, cash, and digital payment solutions such as PayPal, GooglePay, or ApplePay that foster usefulness and/or usability
Perceived limitations of known payment solutions	Perceived negative properties or restrictions of established payment solutions such as credit/debit cards, cash, and digital payment solutions such as PayPal, GooglePay, or ApplePay that decrease usefulness and/or usability
Concerns with known payment solutions	Worrying about consequences when using a particular known payment solution
Perceived equivalence of the digital euro and known payment solutions	Degree to which a digital euro is seen as similar in its positive and negative properties and features as compared to known payment solutions
Perceived benefits of a digital euro	Perceived positive properties of features of a digital euro
Perceived limitations of a digital euro	Perceived negative properties or restrictions of a digital euro
User-specific factors	Context-independent factors of a user of a payment solution
Payment context-specific factors	The specific characteristics of a transaction and payment
Perceived trust	A variety of trust-related factors of users in a payment solution, payment ecosystem, and payment issuer

In the fourth step, the transcribed interviews are coded with preliminary codes to define suitable subcategories. During the coding process, it became apparent that the main category payment context-specific factors and user-specific factors need to be added to our model as they are essential for respondents. In the fifth step, all interviews are being coded again with the final coding guidelines and subcategories to verify that all interviews are coded equally. The last step is the analysis and interpretation of the results. Table 3 in the Appendix provides an overview over some of the total 865 categorized statements.

The interviews demonstrated that respondents indeed explicitly compared the digital euro to known and existing payment technologies and solutions. One rationale for this comparison voiced by several respondents was that they have a hard time understanding, or imagining, a digital euro and the consequences of it. For instance, Expert No. 6 states:

“*So far, I have not actively dealt with this. It’s also possible that a digital euro wouldn’t be much worse than the current payment processing. We simply don’t know enough about the digital euro […]. It’s still a bit of an abstract construct to be able to judge it one hundred percent.*” (Expert No. 6)

The comparison of existing technologies with new technologies, which could not adequately be experienced yet, therefore offers respondents the possibility to form attitudes towards the new technology.

In the following sections, we elaborate on the categories that influence attitudes towards a digital euro.

#### Perceived benefits of established payment solutions

Respondents stated a wide variety of factors and characteristics of established payment solutions that they viewed as beneficial for their payment behavior. Across all payment solutions, ease of use in payments was the most frequently named factor. Respondents hereby draw a comparison between several established solutions, such as cash, credit cards, or digital payment technologies. Frequently, a high degree of convenience, that is high usability, is stated as the main reason of a respondent as the reason for preferring one solution over another. See, for instance, Expert No. 2:

“*So actually, I would say the vast majority is PayPal. I use that in particular because there are many sites, especially if you order something online now, where simply paying with PayPal is the most uncomplicated. I log in and everything is done automatically. I don’t have to wire anything.*” (Expert No. 2)

The wide acceptance of a payment solution is the second most frequently stated perceived benefit. Respondents universally state that cash remains the most widely accepted solution for, low-value, offline transactions. While digital solutions such as PayPal are widely used online, credit or debit cards have the advantage of being also accepted in foreign countries. This demonstrates the influence of payment-specific factors, such as payment amount, type, or purchased product on perceived benefits.

Other perceived benefits are more closely tied to specific payment solutions. For instance, digital payment solutions and credit cards are valued because they offer the possibility to conduct contactless payments. Similarly, these solutions offer a clear overview over ones finances. Past transactions can easily be viewed, clustered, and analyzed by the payee, allowing respondents to remain aware of their purchasing behavior and finances.

Two perceived benefits of cash are of particular interest. Firstly, cash offers the unique option of privacy-friendly, i.e., anonymous, transactions. Although a face-to-face transaction does, under most circumstances, reveal the identity of buyer and seller, no records of the transaction are created and stored. Several respondents referred to cash as an anonymous means of payment and voiced privacy concerns that are discussed in a later subsection in more detail. Secondly, cash was associated with the intangible concept of freedom.

“*[…], but for me, cash is also freedom in action.*” (Expert No. 5)

The rationale behind this is that cash is perceived with a stronger sense of ownership than other payment methods. Fiat money, transferred through credit cards or digital payment methods, is stored in accounts at retail banks. Such bank accounts can be frozen or plundered with the account holder having little power to stop it. Cash on the other hand can be stored in a safe location directly at the owners’ disposal.

#### Perceived limitations of established payment solutions

Respondents frequently regard the perceived benefits of one payment solution as the perceived limitations of another established payment solution. While cash is praised for its wide offline acceptance, in rural areas or smaller stores, it is limited in its usage for online transactions. Conversely, digital payment solutions such as PayPal are often stated to be accepted for digital payments but not offline ones, making the perceived benefits of cash the limitations of digital payment solutions and vice versa. These perceptions are not constant for a specific payment solution across all respondents. For instance, although credit cards are often praised for their convenience, Expert No. 6 regards them as inconvenient in the context of internet payments as the authentication process is perceived as cumbersome due to the two-factor authentication process.

Lastly, respondents described the lack of unique selling proposition as a perceived limitation for payment solutions that they were not using. Respondents stated that they do not need a particular payment solution as they simply have no need for it. They are already satisfied with the options that they know and use.

#### Perceived concerns with established payment solutions

Perceived limitations of established payment solutions are defined as perceived negative properties that a specific solution might possess. Apart from such limitations, respondents also voiced concerns, potentially negative consequences they could face when using a certain payment method. The concerns can be differentiated into security and privacy concerns that are closely linked to a lack of trust. Security concerns were identified to refer to perceived security threats in the technical system and infrastructure of banks and payment systems. Privacy concerns are voiced with respect to payment- and identity-related information which are created or transferred during the payment process.

“*When I think about all the data I give away, especially with credit cards or also with PayPal – so I often use PayPal in e-commerce – that’s data protection. When I consider that they actually know about all my transactions and also what I buy and can basically create patterns of me, like about many other so people. But the transaction data says so much about you*.” (Expert No. 4).

Expert No. 9 recounts that his/her credit card details were stolen once. The stolen information was then used to purchase goods in a foreign country. Expert No. 7 argues that the banking system as a whole is prone to cyberattacks, which leads to general security concerns when using banking solutions.

“*The much worse thing is that today we have a payment system with TARGET2 that is constantly getting cyberattacks and which is simply no longer hack-free and hack-proof. We have banks with outdated IT that can’t handle it. We have phishing, we have scamming. We have huge cyber risks in the system today.*” (Expert No. 7).

#### Perceived equivalence of established payment solutions and the digital euro

Respondents did not solely assess the perceived benefits, limitations, and concerns of existing payment methods in order to transfer them to a digital euro. Instead, respondents assessed the perceived equivalence of established payment solutions with the digital euro. By assessing the perceived equivalence of established solutions and the digital euro, it can be evaluated how well-perceived properties of established solutions can be transferred towards a digital euro. This perceived equivalence is defined as the degree of similarity between different payment methods regarding their benefits, limitations, and concerns to an individual. The higher the perceived equivalence of a payment solution with the digital euro, the better can perceived properties be transferred towards a digital euro. Thus, perceived equivalence acts as a moderator between benefits, limitations, and concerns of established payment solutions and a future digital euro.

“*And if I have the option of paying digitally just as conveniently with central bank money, then I would prefer to do that. Especially if it was also partially anonymous. Then I would have all the advantages of cash, but all the convenience of paying with a card.*” (Expert No. 1).

In the first sentence, the respondent states the perceived benefit of digital payment solution, a high degree of convenience. In the second sentence, an assessment on the perceived equivalence of cash and digital euro transactions is drawn. The respondent then concludes that a digital euro could possess all the advantages, perceived benefits, of cash, as well as one perceived benefit, convenience, of card payments. The digital euro is therefore assessed through the individual evaluation of other payment methods. Naturally, a low or negative equivalence of payment solutions leads to contrary results in the respondents’ assessment. The absence of a perceived benefit of cash, for instance, leads to a perceived limitation of the digital euro.

Such relationships between payment systems, moderated through a perceived equivalence, are found for three payment solutions, namely credit/debit cards, cash, and digital payment methods.

“*If the user interface is really like it is right now with PayPal or credit card then yes. It should be as easy to use as possible.*” (Non-expert No. 10).

“*I can also imagine that. If it’s actually easier than paying with a checking card, because you can see everything in your digital wallet. Maybe you’ll also have access to how much you still have in your account and so on. And then it might be much more practical than paying with a checking card.*” (Non-expert No. 9).

It is noted that CBDCs are not compared to cryptocurrencies by respondents.

The comparison of payment solutions is influenced by additional factors such as a feeling of trust that led to expectations on how a digital euro should be. Specifically, trust in the central bank leads respondents to believe that the digital euro is to be more privacy-sensitive than other payment solutions:

“*If it is issued by the ECB, then expect more data protection and more anonymity, or the possibility to pay more anonymously, than with a debit card.*” (Expert No. 1)

#### Perceived benefits of a digital euro

The perceived benefits of a digital euro are largely similar to those of established payment methods. Perceived ease of use, convenience and high usability, and performance expectancy, quick and cheap transactions, are the factors most often mentioned. Respondents argue that a digital euro can only be of use if it is widely accepted, by other individuals as well as merchants and organizations, which depends on how easy it is to pay with a digital euro.

“*It is a question of acceptance. So, I probably won’t get myself 5000 digital euros if there’s exactly one store on the Internet where I can pay with it. That would make little sense.*” (Expert No. 3)

The digital euro is also evaluated for different payment use cases, such as peer-to-peer payments, business transactions, or business-to-consumer transactions. Expert No. 3 did not only argue that merchant acceptance would constitute as a perceived benefit but argues that acceptance by different stakeholders can be difficult to achieve. Experts No. 1 and 6 state that programmability and machine-to-machine payments would constitute additional benefits although they might not be of use for retail payments.

Respondent No. 8 describes the perceived benefit of holding money directly at the central bank, offering stability and a stronger level of protection in terms of crisis, as compared to money stored at retail banks.

“*I want to say again why this sounds attractive to me. There is of course the stability of it [the digital euro] compared to money I have in a bank. A bank that could very well go bankrupt.*” (Non-expert No. 8)

Lastly, the opportunity for anonymous transactions is seen as a potential unique selling point of a digital euro by multiple respondents.

“*[…] that the digital euro is anonymized within a regulatory framework. At least up to a certain amount limit. Then I would see a unique selling point for the digital euro*.” (Expert No. 4)

#### Perceived limitations of a digital euro

As with perceived benefits, perceived limitations are highly diverse and largely based on respondents’ perceived limitations of other, established digital payment solutions. Some factors that were stated as perceived benefits by some are classified to be perceived limitations by others.

A main limitation of a digital euro was clearly voiced by respondents as the lack of added value over existing payment solutions. Respondents stated that they are satisfied with their current selection of payment solutions:

“*As long as it was newly introduced, I wouldn’t use it for the time being, because I personally wouldn’t get any added value from it. The payment service providers I currently use are simple and straightforward to me. I don’t see any added value as to why I should suddenly use something else.*” (Expert No. 2)

“*I don’t yet see the advantage over classic online banking. So, if I simply use [digital payment provider 1] or this [digital payment provider 2] or something like that on the Internet, then that’s also done very quickly.*” (Non-expert No. 7)

Another related point is also raised by multiple non-experts. The digital euro is described as being intangible; the concept of it and how exactly it would differ from other digital payment methods is not understood. The market of payment solutions is saturated, and respondents do not see how a digital euro could possess a unique selling point.

“*I think we already have digital payment methods. I can pay digitally at any bank, mostly with my cell phone. There’s ApplePay, GooglePay, AmazonPay and all sorts of other things. So, we have to ask ourselves, and the ECB should ask itself: do we even need a new digital payment method? Because actually the market is saturated.*” (Expert No. 1)

Several limitations could not be categorized into wider categories and were only mentioned by single respondents. These include the perceived limitation and question as to whether a digital euro could only be used in the euro zone and how it could be converted into other currencies.

With regard to usability, respondents state that filling a wallet up with digital euro could be as cumbersome as using an ATM to withdraw cash.

#### Concerns towards a digital euro

As with established payment methods, the two types of concerns most frequently discussed were privacy- and security-related concerns. This finding is in line with prior qualitative research on user perceptions related to innovative technologies (Harborth & Pape, 2019) and relates to the factors that respondents valued in the ECB’s public consultation (European Central Bank, 2021).

Security is even seen as a prerequisite for the intended usage of a digital euro, as stated below.

“*It always depends on the security. Because if it’s created digitally, it can also be removed digitally. I have to get to know this security mechanism and if I know that it works one hundred percent and nothing can go wrong.*” (Non-expert No. 6)

There are two primary types of perceived security concerns grounded in the data. The first one relates to the perceived security of the user-facing technology (the wallet or account which holds the digital euro). Individuals fear losing access to their account or not being able to properly protect their wealth because of unauthorized access and theft.

“*The digital euro needs a corresponding wallet. Many people today are negligent with their passwords, their smartphones, etc. I think security is a big issue here.*” (Expert No. 9)

“*No, it’s more about abusive access and, let’s say, digital theft*.” (Expert No. 9)

The second perceived security concern relates to the underlying technology and the interplay of systems and organizations that might lead to a loss of security. The technical implementation of a digital euro therefore comes with security risks that cannot be adequately evaluated yet.

*[…] security has a lot to do with people and processes and a world that’s not fully digitized. But for me, the more I understand how it works, the more I have to frown at times that it works. Because it all possesses an extreme number of moving parts [the interplay of entities and technologies in financial transactions]. When I kind of break it down to just the technology stack, I let myself believe that there’s a greater understanding that leads to a greater sense of security. But I think the moment we look at the periphery, that can also change. Because it’s not just about the payment protocol. It’s also about the apps we use to operate it. It’s about the terminals we then use to identify ourselves somehow. It’s probably about how we, as individual people, how we authenticate ourselves against this system, so to speak, where there are passwords lying around on paper and so on*. (Expert No. 8).

Privacy in digital euro transactions was discussed by all respondents. Concerns about the control of personal information are the most frequent stated first-order dimension of privacy concerns. Thirteen out of 16 interviewees who raised privacy concerns with regard to themselves or a third party were concerned about losing control over personal data. Other dimensions are concerns about the amount of data collected (collection) and about unauthorized access (improper access).

#### Payment context- and user-specific factors

When discussing established payment solutions, payment context-specific factors were found to increase or decrease perceived benefits, limitations, and concerns towards these solutions, as stated by respondents. Depending on the context of a payment, the benefits of a payment solution may be more pronounced, or concerns with the solution may be enhanced.

We identified five different types of payment context, namely the payment purpose, the involved entities, transaction sensitivity, frequency, amount, and the scope of the transaction.

For transaction purpose, respondents were found to distinguish business to business (B2B), business to consumer (B2C), and peer-to-peer (P2P) payments. As the focus of the interviews has been the evaluation of the digital euro as a currency for retail use cases, B2B payments were not specifically questioned. Yet, respondents already evaluated possible limitations and benefits in the business context.

Particularly privacy and security concerns were found to relate to the entities involved in a payment process, the sensitivity of the transaction, and the transaction amount. Respondents want sensitive transaction or product information to remain private from family members or state agencies. Lower value transactions were frequently described as being less privacy-sensitive than high-value transactions. A digital euro would offer higher benefits if it could be used for online and offline transactions as well as for national and international ones. Furthermore, respondents assigned different payment solutions towards payments of specific frequency. Infrequent transactions, which are often high-value transactions, were often conducted using credit cards, while frequent, low value, transactions were often conducted using cash.

#### User-specific factors

User-specific factors describe factors that were found to influence respondents’ attitudes towards a digital euro without being related to specific payment methods. Demographics make up the most frequently identified factor. Further factors are technical and financial affinity, which are also linked to age by respondent. Older individuals may lack the technical knowledge to use a digital euro or digital payment methods in general. Similarly, individuals who lack financial knowledge may not be able to adequately assess the functioning of a digital euro. Age is the factor most frequently cited to influence payment habits towards cash. Naturally, experts stated that their professional background influences their perception on the digital euro, e.g., through their assessment on data protection or cybersecurity. Non-expert No. 6 referred to homeless people and how they would be able to use a digital euro.

These findings demonstrate that attitudes towards different payment solutions are influenced by individuals’ professions as well.

“*I can imagine that many people have exactly this thought that they will lose their cash—many people are still attached to cash, I think—and everything will become digital. Older people in particular, perhaps.*” (Non-expert No. 5)

Respondents furthermore pointed out the impact of personality- or identity-related factors on attitudes towards payment solutions. Specifically, cultural upbringing and ones’ personality were mentioned. The German population is frequently classified as being highly privacy-sensitive and in favor of cash usage. These points were made from both the first- and third-person point of view. For the interviews, experts were asked to not only state their personal assessment but to also discuss the topic based on their professional assessment, using a third-person point of view.

## Discussion

The results demonstrate that the EAFT is applicable to the digital euro. Perceived benefits, limitations, and concerns of established payment solutions are influencing the attitudes towards a digital euro, mediated by the perceived benefits, limitations, and concerns of the digital euro. The relationship between these perceptions is moderated by the perceived equivalence of the respective payment solution in comparison and the digital euro. Respondents did not consistently express the same benefits or limitations for a payment solution. For instance, a respondent may attribute a high level of ease of use towards digital payment methods, whereas another respondent may regard this as a perceived limitation of digital payment methods. Participants are heterogeneous and do not gain the same utility from a technology. However, perceived benefits of a digital euro universally do have a positive impact on attitudes towards a digital euro, and perceived limitations and concerns have a negative impact on attitudes towards a digital euro. As the great majority of respondents have received some form of higher education, this strong educational background needs also is regarded as part of the user-specific factor. Education could therefore influence the assessment of perceived benefits, limitations, and concerns. One example could be the importance of privacy in digital euro payments, as prior research found that higher education is associated with higher privacy concerns (Sheehan, 2002).

User- and payment context-specific factors explain these differing assessments as they, in turn, influence perceived benefits, limitations, and concerns towards established payment solutions. For instance, the ability to pay in foreign countries using credit cards is a stated perceived benefit of card payments that is especially useful for individuals that frequently travel internationally for their job (user-specific factor) and frequently conduct payments (payment context-specific factor) abroad.

Here, cultural preferences and ideological factors also play an important role. Several respondents associated cash payment with the notion of “freedom” and elaborated on the German preference for privacy. There likely is a limited perceived equivalence between cash and a digital euro for these factors. However, respondents compared the payment methods that they are currently using with each other and elaborated on their “optimal” digital euro by combining their preferred features or benefits. A central bank could greatly improve users’ attitudes towards a CBDC should it be able to create a CBDC while considering these factors.

To this end, the perceived benefits of cash, freedom in transaction and privacy, could be combined with the perceived benefits of digital payment solutions such as wide acceptance or ease of use. One respondent outlines this by stating:

*My opinion is that they [the ECB] have to create a certain USP that makes the digital euro stand out from all other digital payment methods. And because it is the ECB and the ECB is the only one allowed to issue central bank money, it has to be an advantage of cash*. (Expert No. 1).

Security and privacy concerns will need to be overcome, through technological transparency, regulation, and the fostering of trust in central banks, the state, and the monetary system. Such security privacy concerns or risks were also identified to be influencing technology adoption in cryptocurrencies and can be transferred from cryptocurrencies to other digital payment service providers such as PayPal (Fota et al., 2022).

When discussing the crucial factor trust, Smith et al. (2011) note that the factor is modeled differently across various studies. Trust is modeled as a mediator between privacy concerns and outcomes (Alashoor et al., 2017), as an antecedent for privacy concerns, perceived benefits, and perceived risks (Li et al., 2014), as well as an antecedent for behavioral intentions (Dinev & Hart, 2004). In this work, the variable trust is found to be included in the categories of perceived benefits, limitations, and concerns of established payment solutions and the digital euro. Through the content analysis, we identified that respondents discussed multiple trust dimensions in different contexts. For instance, Expert No. 7 mentions trust in the state and the ECB, a perceived benefit of the digital euro.

“*I also don’t have the feeling that the state will steal from me tomorrow. I’m on board with the digital euro, that’s because of the ECB’s policy.*” (Expert No. 7)

This trust in the central bank corresponds to a distrust in retail banks (perceived limitation of established payment solutions) which was also voiced as a concern when using digital payment solutions.

“*Because I trust a government agency significantly more. With the ECB, you just know there are government control mechanisms. They are bound by certain market standards and things and can’t just do what they want. If you go to private banks, it’s completely non-transparent. Of course, there are certain transparency mechanisms, but it is still up to the private company to decide what to do with the profits and your money. And that’s not the case with the ECB.*” (Expert No. 6)

Similarly, the creation of trust in CBDC will differ from that in cryptocurrencies given that many factors for trust in cryptocurrencies such as decentralization, immutability, and the opportunity to use it as an investment (Marella et al., 2020) are not present in CBDC. For CBDC, Bijlsma et al. (2021) also differentiate between different types of trust, including *narrow-scope trust* in a individuals’ own retail bank, *broad-scope trust* in banks in general, *trust in the central bank*, as well as *generalized trust* that other individuals will be using CBDC. These differentiations were also found in our work, as respondents differentiated explicitly between different institutions and organizations when talking about trust, citing a lack of regulation or supervision as well as business incentives as reasons for distrust in retail banks and other financial service providers. Thus, compared to existing technology adoption models such as TAM or UTAUT, which could not demonstrate a definitive way to include trust in the model, our adapted model offers the advantage that trust is included in the perceived benefits, limitations of concerns of respondents.

These findings however need to be interpreted in the context of this research, on a digital euro with German, highly educated, respondents. Trust in central banks is comparably strong in advanced economies such as Japan, France, the USA, and Germany (Patel & Ortlieb, 2020). Emerging markets, such as Russia or India, demonstrate much higher degrees of trustworthiness for central banks as well as for retail banks, other payment service providers, and big tech companies. Such other organizations are generally regarded as untrustworthy in European countries (Patel & Ortlieb, 2020). Trust in the ECB is comparable between European countries (Bursian & Fürth, 2015). The German average level of trust in ECB differs between Eastern and Western Germany (Angino et al., 2021) but remains between that of France, with a lower average level of trust, and the Netherlands, Ireland, and Portugal, with a higher average level of trust in ECB (Bursian & Fürth, 2015; Cruijsen & Samarina, 2021).

Furthermore, our results indicate that privacy concerns in particular are of a key factor when discussing user attitudes towards a digital euro. These findings are in line with the previous report of the ECB in which respondents, especially from Germany, chose privacy in payments as their most important feature in a digital euro (European Central Bank, 2021). Prior research indicates that German citizens demonstrate a particular preference for privacy protection (Krasnova & Veltri, 2010) and privacy regulation (Dogruel & Jöckel, 2019) when compared to other nationals such as US citizens. While privacy is seen as the most important factor in a digital euro among most respondents, 55% of German respondents ranked it most important, with all other European countries, such as the Netherlands (about 48%), demonstrating a less strong preference for privacy. Moreover, as respondents from different countries demonstrate different preferences for payment methods, their attitudes towards these payment methods might differ. Differences in preference for cash are nonetheless decreasing with a strong push towards digital payments, among Europe, following the COVID-19 pandemic (Mai, 2021). With regard to preferences for payment methods in general, it can be seen that there are differences between countries. For instance, the online banking e-payments service iDEAL has a 71% market share in the Netherlands (iDEAL, 2021), while many other European citizens prefer cards for digital transactions (Mai, 2021). Germany hereby deviates from other countries with both a high preference for e-payment solutions such as PayPal and a preference for credit transfers. Moreover, as the sample consisted of relatively young and higher educated individuals for both experts and non-experts, elderly and less educated individuals’ attitudes might differ from the ones identified in this work. Comparable research on attitudes and adoption of past payment innovations such as credit cards and ATMs demonstrate similar relationships (see Hirschman and Julander, 1979; Awh & Waters, 1974; Swinyard & Ghee, 1987). Here, habits, perceived risks, as well as demographic factors are also found to influence attitudes and adoption intention (Murdock & Franz, 1983; Swinyard & Ghee, 1987).

With these differences in preferences in mind, the general framework of the attitude formation model for CBDC would however remain the same as the category of “user-specific factors” accounts for these differences in demographics and culture. This factor could nonetheless influence attitudes towards CBDC, as, for instance, the perceived equivalence of CBDC and credit card payments could be strongly perceived as negative if CBDC payments would not allow the delay of payments using CBDC, a feature that credit card payment offer.

The main contribution of this work is however not the finding that attitudes towards a digital euro are indeed influenced by attitudes towards other payment methods, but rather the insights that can be drawn from this comparison. Respondents indicated that the perceived equivalence of a digital euro is highest with other digital payment methods and rather low with cash. This makes sense as the digital euro is imagined to be used for digital payments and to complement cash (European Central Bank, 2020). However, several respondents state that they see no immediate use for CBDC in general as they are satisfied with their current payment methods and praise digital payment methods and card payments for their ease of use and widespread adoption. They do however also discuss perceived limitations of existing digital payment methods. In order to find a niche in this saturated market, in which a digital euro can not only survive but thrive, it is necessary to eliminate the negative characteristics of competitors in the development of the digital euro. This needs to be done under consideration of payment context- and user-specific factors, as well as the requirements and goals that the ECB has for a digital euro. One crucial point-of-access to the payment market could be privacy. Respondents state that privacy-friendliness could be a factor that can act as a strong perceived benefit for the digital euro that could act as a competitive advantage. As of now, cash remains the most privacy-friendly mean of payment, which is regarded as a substantial perceived benefit of cash by respondents, while digital payment methods store and analyze large amounts of data that could be processed by untrustworthy entities. It would be highly advantageous to adopt this benefit for the digital euro that aims to supplement cash for digital payments. While the ECB is found to be a generally trustworthy entity by respondents of this work, and in prior research (Bursian & Fürth, 2015), some respondents raised the issue of a power accumulation in central banks through the introduction of CBDC. Fostering trust in the development of a privacy-friendly digital euro could prove an effective strategy as prior work on CBDC demonstrated that trust could mitigate privacy concerns (Tronnier et al., 2022).

Overall, the contribution of this work, the verification that attitudes towards CBDC are formed also through attitudes towards existing payment methods and the development of a conceptual model for this attitude formation process, can be used to create a CBDC that would provide benefits to its users by focusing on the elimination of concerns and limitations of existing solutions against which a CBDC will have to compete.

### Limitations

The main limitation of this work results from the fact that the digital euro does not exist yet, its features and design are not known by respondents at this point in time. Attitudes towards a digital euro, in the form of perceived benefits and limitations, are therefore subject to possible change. New features may, for instance, lead to additional perceived benefits by respondents. This limitation however does not change the overall attitude formation model for CBDC but may only influence the importance of specific factors on the attitudes towards a digital euro in the future.

Moreover, the findings are of limited generalizability as the research was conducted with German participants only of which more than 90% received some form of higher education. Therefore, the results of this work need to be understood in the context of the German population, which demonstrates, for instance, a higher preference for cash usage and a stronger preference for privacy than other countries (Dogruel & Jöckel, 2019; Krasnova & Veltri, 2010), while trust levels among European nations in the ECB and the banking sector are comparable (Bursian & Fürth, 2015; Patel & Ortlieb, 2020). Similarly, higher education might have also impacted attitudes, as again, higher education is associated, for instance, with stronger privacy concerns (Sheehan, 2002). Young and highly educated individuals are however more likely to be early adopters of new technology and could therefore also be the ones more open towards CBDC, representing a good approximation of early adopters. We therefore argue that the results are of value for CBDC developers as those individuals are likely to be the ones to first try using CBDC. However, it is possible that less educated and older respondents might demonstrate differing attitudes, or feel unable to form attitudes towards CBDC, as was observed with two older respondents which could not be included in the sample as they felt unable to discuss the topic. It could also be that their attitudes might differ from the respondents of this work. These differences in demographic factors are however included in our model in the form of the user- and payment context-specific factor. Individuals would therefore simply evaluate the parameters differently, i.e., by strongly valuing perceived benefits of credit card payments such as the ability to delay the payments, that would then influence attitudes towards a future CBDC. Lee and Baskerville (2003) discuss generalizability, in particular for qualitative research, in detail and argue that there exist different forms of generalizability next to sampling-based generalizability. The authors argue that “as a consequence of Hume’s truism, a theory may never be generalized to a setting where it has not yet been empirically tested and confirmed” (Lee & Baskerville, 2003, p.241).

### Future work

Our work provides several directions for future research. Our model needs to be validated through quantitative analysis, preferably using the example of existing CBDCs. Given the geographic focus of this work, similar studies could be conducted in other countries, in the euro area or with other CBDCs. Particularly studies on the latter could provide surprising results as CBDCs may differ between countries given the diverging approaches and objectives of central banks worldwide. Finally, this work acts only as a first step towards a more fine-grained analysis on the factors that ultimately influence adoption intention or actual use and make up perceived benefits, limitations, and concerns of a digital euro.

## Conclusion

This work is, to the best of our knowledge, the first to investigate user attitudes towards CBDCs. We argue that existing models on technology adoption, such as UTAUT or TAM, cannot be applied to CBDC yet as most CBDCs worldwide have not been issued to end users yet and communicated design features and technical solutions differ significantly from central bank to central bank, making it impossible for end users to reliably evaluate the factors used in such models. In this work, we overcome this issue by studying attitudes, the predecessors of adoption intention for technology (Ajzen & Fishbein, 1977). By employing the attitude formation theory (EAFT) by Harborth and Kreuz (2020) as a theoretical underpinning, we obtained an understanding about how individuals form attitudes towards a digital euro by assessing attitudes towards related payment solutions and technologies as well as their perceived equivalence with the digital euro. EAFT proclaims that attitudes towards a new technology, that users have not yet experienced, are influenced by attitudes towards comparable solutions with which users already have experienced. We find that user-specific factors, such as demographics and cultural upbringing, and payment context-specific factors, such as involved entities and transaction amount or frequency, are also of importance in the attitude formation process in our scenario.

As a theoretical contribution, we are the first to verify that the EAFT can indeed be applied to technologies other than augmented reality and add additional factors to it. Based on the EAFT, we develop an attitude formation model specifically for CBDC.

As a practical contribution, this work offers a blueprint on how to best create a CBDC that will be used by its intended users through the analysis of attitudes towards established payment solutions and the transfer of these attitudes. Several participants indicated that they currently see no reason for using a digital euro as an additional payment solution given the current benefits of established payment solutions. However, existing payment solutions are also found to possess perceived limitations and concerns by respondents. A CBDC needs to obtain a competitive advantage over existing solutions through the combination of perceived benefits of other payment methods while eliminating their limitations and mitigating users’ concerns. More specifically, perceived limitations and concerns of related solutions, such as low usability of cash in online settings and privacy concerns and trust issues towards digital payment solutions, can be seen as target factors through which a digital euro might obtain a competitive advantage. Thus, a digital euro would need to be privacy-sensitive, offering wide acceptance for national and international online and offline payments in different contexts.

Respondents voiced that they would potentially approve of such a one-size-fits-all solution, given their current usage of multiple payment solutions in different circumstances and for different use cases. However, central banks need to evaluate whether such a CBDC is actually technologically feasible and strategically advisable taking into consideration the current and future regulatory framework and the central banks’ monetary objectives with said CBDC.

## Data Availability

The participants of this study did not give written consent for their data to be shared publicly, so due to the sensitive nature of the research supporting data is only available upon reasonable request from the corresponding author.

## Appendix

Table 3

**Table 3:** Extracts on the codings of all statements

Statement no	Interview	Primary code	Subcode	Person point of view	Segment
33	E1	Perceived concerns with credit cards	Privacy	1st	And I think the most important and exciting advantage of cash is anonymity. And that’s also the reason why people who don’t pay much by card still pay with cash
68	E10	User-specific factors	Demographics	1st	Then I think if there is a wallet-only solution and not an additional parallel solution on a hardware token or something like that, I think we would have difficulty bringing the elderly population along in the change
69	E10	Perceived limitations of digital euro	Need for internet	1st	Furthermore, I believe that Internet coverage is a challenge in Germany
75	E10	User-specific factors	Culture	3rd	And I also believe that Germans are not as open to new technologies as other countries. I think they are skeptical at first. And they also have the reputation, but everything has to be perfected first before you publish anything and I think that also plays a role…
195	E5	Perceived benefits of digital payments	Cost	1st	Once the online banking of course because of the convenience, and also, that is a matter of cost nowadays
196	E5	Payment context-specific factors	Transaction amount	1st	I consciously use cash for everyday things, but of course I always have cards available in my wallet. That is, if it goes beyond, let’s say, a normal cash amount, then of course, you also use card payment
248	E6	Payment context-specific factors	Transaction purpose	1st	I have to say that I differentiate very strongly according to the intended use
259	E6	Perceived equivalence of established payment solutions and the digital euro	CBDC—Credit Card	1st	So, I think for me, I would consider using it if those disadvantages of the giro payment process that I just described, that is, the digital trail that you leave behind, if that would go away. And then it would actually be a very attractive option for me compared to cash
265	E6	Perceived limitations of digital payments	Trust	1st	If you work for private banks, it’s completely non-transparent. Sure, there are certain transparency mechanisms, but it’s still up to the private company to decide what to do with the profits and your money. And that’s not the case with the ECB
270	E6	Perceived benefits of digital euro	Ease of use	1st	For me, the digital euro would be very customer-oriented, i.e., actually similar to all these pay services like ApplePay or something like that. In other words, it would be as uncomplicated as possible to use
363	E9	Perceived concerns with credit cards	Security	3rd	His [A friends] EC card details have been read and used to make purchases at Harrods in London
364	E9	Perceived benefits of digital payments	Transaction speed	1st	… online banking and cashless payments are also simply a matter of convenience and also transaction speed for me
470	NE1	Perceived equivalence of established payment solutions and the digital euro	CBDC—Cash	1st	I mean, that would actually be even simpler than cash, because there’s no change and all that. So, in principle, yes, if it’s somehow simple and secure, then by all means
499	NE10	Perceived benefits of cash	Acceptance	1st	I’ve been in [city] forced to [use cash], but I really only use cash if I have to, if they don’t take a card
516	NE10	Payment context-specific factors	Payment purpose	1st	Personally, I don’t think I would ever prefer that. The only situation would be if my grandma just slips me a little cash—then I wouldn’t refuse it
714	NE7	Perceived limitations of cash	Perceived limitations	1st	and that [cash] goes always empty, so that I have to regularly search for an ATM
807	TE2	Perceived concerns of digital euro	Privacy	1st	I assume the transactions are most likely traceable. But there are probably ways to change that. But I do not know what the concept looks like – that is one point
811	TE2	Perceived concerns of digital euro	Privacy	1st	To allow as few conclusions as possible, because I think that is my personal business
816	NE8	Perceived concerns of digital euro	Privacy	1st	As a person to whom privacy is very important, I want to leave as little traces as possible behind with regard to my behavior
847	NE8	Perceived concerns of digital euro	Privacy	1st	Traces revealing my consumption patterns, in particular, can be used to place more relevant adverts for me
855	NE7	Perceived concerns of digital euro	Privacy	1st	Only the service provider and I will know about it. I think that having this option is important to me
